# Cl:Na ratio on ICU admission as a prognostic indicator of mortality in sepsis patients

**DOI:** 10.1186/cc13420

**Published:** 2014-03-17

**Authors:** HK Atalan, B Gucyetmez, N Cakar

**Affiliations:** 1 Atasehir Memorial Hospital, Istanbul, Turkey; 2 International Hospital, Istanbul, Turkey; 3 Istanbul Medical Faculty, Istanbul, Turkey

## Introduction

The aim of the study is to investigate the relationship between Cl:Na ratio disturbances and mortality in sepsis patients. Stewart's strong ion theory can be used in the interpretations of acid- base abnormalities [1]. The Cl:Na ratio is a simple alternative test that obviates the need to solve the complex strong ion gap (SIG) equations[2].

## Methods

A total of 434 sepsis and severe sepsis patients hospitalized in the ICU of two centers between 2006 and 2012 were included in the study. Three groups were formed as patients having low (<0.75), normal (>0.75 to <0.80) and high (<0.80) Cl:Na ratio within the first 24 hours in the ICU. Patients' age, gender, APACHE II score, SOFA score, pH, PaCO_2_, HCO^3^, base excess, Na, K, Cl, Ca, lactate, strong ion difference, anion gap, length of ICU stay and mortality were recorded. Logistic regression analysis was used to calculate odd ratios and 95% CIs for the association of Cl:Na with mortality. In the fully adjusted model, pH, BE, AG, lactate and Cl:Na ratio were entered into the model. *P *< 0.05 was considered statistically significant.

## Results

**Figure 1:**
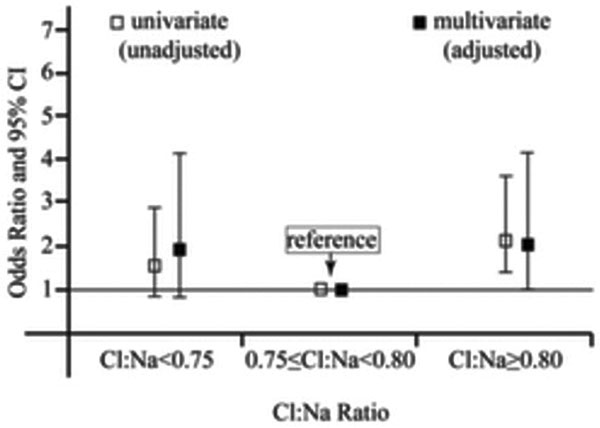
**In the multivariate model, effect of Cl:Na ratio group was adjusted for pH, lactate, anion gap and base excess**.

The distribution of the patients was as follows: low Cl:Na (75, 17%), normal Cl:Na (243, 56%), high Cl:Na (116, 27%). Univariate analysis revealed that in low and high Cl:Na ratio patients, mortality was higher by 1.56-fold (0.87 to 2.81) and 2.22-fold (1.36 to 3.61) (*P *= 0.135 and *P *< 0.01). In multivariate analysis, increased mortality by 1.95-fold (0.92 to 4.12) and 2.02-fold (1.01 to 4.03) was found in low and high Cl:Na ratios (*P *= 0.081 and *P *= 0.046) (Figure 1).

## Conclusion

Stewart's strong ion theory provides assessment for the etiology of acid-base disturbances. This evaluation can be performed easier and faster with the Cl:Na ratio. This study demonstrates that the disturbed Cl:Na ratio is associated with increased mortality in sepsis and severe sepsis patient groups.
